# Supplementary material for: Contextual cues are not unique for motor learning: Task-dependant switching of feedback controllers
Source: PLoS Comput Biol. 2022 Jun 9;18(6):e1010192. doi: 10.1371/journal.pcbi.1010192 (PMC9217135; doi:10.1371/journal.pcbi.1010192)
Supplement: S1 Text — Here we verify whether our participants developed different feedback controllers for hit and stop tasks over the blocked schedule, or if these controllers were innate. To do so, we analyse the visuomotor responses in the first few blocks of the study, showing that these responses can be considered innate. (PDF) [file pcbi.1010192.s001.pdf]

S1 Text. Initial learning of feedback controllers

One of our main goals in this study was to test whether participants could or could not rapidly switch between two feedback controllers. As a result, we selected two tasks (stop and hit) that we expected participants would have innate controllers for. However, it is possible that these controllers were not in fact innate, and participants only developed them over the extended exposure to different tasks during blocked schedule. We therefore analysed the feedback responses of our participants over the first three blocks of each condition qualitatively (Fig S1). Even within the early portion of the experiment, we observe a clear distinction between feedback responses in hit and stop conditions, suggesting that controllers were indeed innate to our participants, or at least developed rapidly enough to be considered as innate for the purposes of our study.

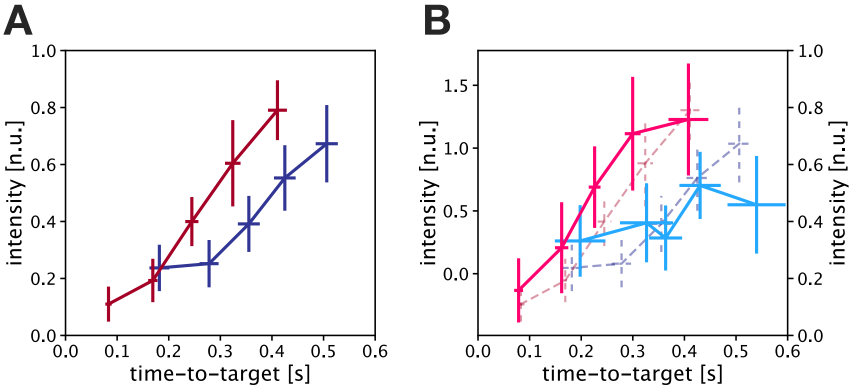

Fig S1. Visuomotor feedback intensities during the first three blocks of the experiment. **A.** Normalised feedback intensity profiles of participants in blocked stop and blocked hit conditions, expressed against time-to-target. Participants produce stronger responses at matching time-to-target in the hit condition, consistent with simulation results for hit and stop (same as Fig 3E). **B.** Normalised feedback intensity profiles of participants in blocked stop and blocked hit conditions during the first three blocks of each condition, expressed against time-to-target (solid lines, primary axis). Dashed lines represent the responses over the entirety of the blocked schedule (secondary axis). Error bars represent 95% CI
