# Supplementary material for: Contextual cues are not unique for motor learning: Task-dependant switching of feedback controllers
Source: PLoS Comput Biol. 2022 Jun 9;18(6):e1010192. doi: 10.1371/journal.pcbi.1010192 (PMC9217135; doi:10.1371/journal.pcbi.1010192)
Supplement: S2 Text — Visuomotor responses were analysed in trials immediately following the condition switch (hit to stop or stop to hit) in mixed schedule. Analysis shows same regulation as in the entirety of the mixed schedule, implying rapid switching. (PDF) [file pcbi.1010192.s002.pdf]

S2 Text. Effect of condition clustering in mixed schedule

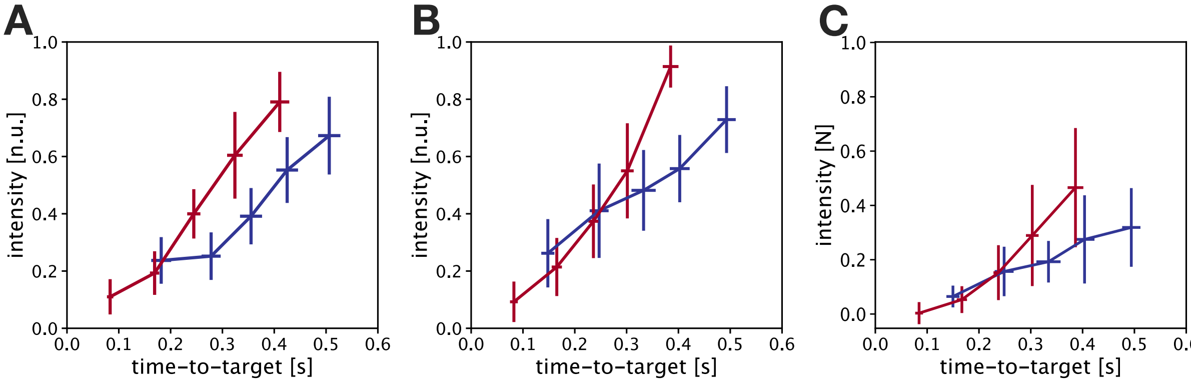

Fig S2. Visuomotor feedback intensities in the trials after the switch of condition. **A.** Normalised feedback intensity profiles of participants in blocked stop and blocked hit conditions, expressed against time-to-target. Participants produce stronger responses at matching time-to-target in the hit condition, consistent with simulation results for hit and stop (same as Fig 3E). **B.** Normalised feedback intensity profiles of participants in mixed stop and mixed hit conditions, only in trials immediately after condition switch, expressed against time-to-target. Similar regulation is observed as in the blocked schedule. **C.** Raw feedback intensity profiles of participants in mixed stop and mixed hit conditions, only in trials immediately after condition switch, expressed against time-to-target. Error bars represent 95% CI

In our study design we took deliberate care in shuffling the conditions in mixed schedule. Particularly, if we simply shuffled the 832 trials in the mixed schedule together, we are likely to observe large clusters of the same condition without switching, which may allow for adaptation of feedback controllers and thus would provide false support for the rapid switching hypothesis. In order to avoid such effects we used a pseudo-random design, where we divided the 832 trials in the mixed schedule into 26 consecutive blocks of 32 trials each. Every block contained 16 trials of hit condition, and 16 trials of stop condition, where the 16 trials contained 11 perturbation trials (5 perturbation onset locations $\times$ 2 directions, plus one zero-perturbation trial) and 5 free (null-field) trials. Within each block all trials were randomly shuffled, but one block had to be completed entirely before the next block started. Similar shuffling was also performed in the blocked schedule, only that each block contained 16 trials of a single condition, and all 26 blocks of one condition had to be completed before the opposite condition was first introduced.

Even under the aforementioned constraints of the randomisation there still remains a chance of condition clustering. In fact, on average in the mixed-schedule each participant experienced 52% of trials after condition switch, with remaining trials following the same condition as on the previous trial (26% after exactly one trial of the same condition, 12% after exactly two trials, and the larger clusters in diminishing quantity). As a result, we performed a control analysis where we compared the hit and stop conditions only in trials immediately after the condition switch (Figure S2). A one-way ANCOVA analysis of normalised visuomotor response intensity in the mixed schedule with condition as a fixed factor and time-to-target as the covariate still showed a significant main effect of condition (hit or stop, $F_{1,127} = 5.51$, $p = 0.02$), despite reduced statistical power. However, a similar Bayesian ANCOVA analysis only showed a weak tendency towards main effect for condition ($BF_{incl} = 1.96$). Both analyses showed a significant effect of time-to-target ($F_{1,127} = 94.3$, $p \ll 0.001$; $BF_{incl} = 2.86 \times 10^{1}3$). Thus, qualitatively and quantitatively the control behaviour of our participants during the first trials after condition switching matched that demonstrated for the whole

duration of the experiment, showing no effect of learning due to consecutive trials of the same condition. As this analysis uses only a subset of our data, these results are sensitive to noise, and thus the normalisation of the data, shown in Figure S2B could be affected by an occasional outlier. As a result, for completeness, and a clearer picture, present both normalised data (S2B), and raw data (S2C).
