# Supplementary material for: Contextual cues are not unique for motor learning: Task-dependant switching of feedback controllers
Source: PLoS Comput Biol. 2022 Jun 9;18(6):e1010192. doi: 10.1371/journal.pcbi.1010192 (PMC9217135; doi:10.1371/journal.pcbi.1010192)
Supplement: Attachment — Submitted filename: Reviewer_responses.pdf [file pcbi.1010192.s004.pdf]

Dear Mr Česonis,

Thank you very much for submitting your manuscript "Contextual cues are not unique for motor learning: Task-dependant switching of feedback controllers" for consideration at PLOS Computational Biology.

As with all papers reviewed by the journal, your manuscript was reviewed by members of the editorial board and by several independent reviewers. In light of the reviews (below this email), we would like to invite the resubmission of a significantly-revised version that takes into account the reviewers' comments.

Overall, the reviewers were generally positive about the paper and found the paper to be technically sound. Reviewer 2, however, raised an important issue about the novelty of the work, pointing out several papers that have already provided evidence for the general claim that people are able to flexibly switch between different controllers in a task-dependent manner. I believe (and the reviewer seems to agree) there is sufficient novelty in the current paper to warrant publication. However, I agree with Reviewer 2 that the paper needs to better acknowledge previous studies that have already addressed the general question of switching between feedback controllers (and reached the same conclusion), and should more clearly distinguish the specific contribution of this paper in relation to that prior work.

We cannot make any decision about publication until we have seen the revised manuscript and your response to the reviewers' comments. Your revised manuscript is also likely to be sent to reviewers for further evaluation.

When you are ready to resubmit, please upload the following:

[1] A letter containing a detailed list of your responses to the review comments and a description of the changes you have made in the manuscript. Please note while forming your response, if your article is accepted, you may have the opportunity to make the peer review history publicly available. The record will include editor decision letters (with reviews) and your responses to reviewer comments. If eligible, we will contact you to opt in or out.

[2] Two versions of the revised manuscript: one with either highlights or tracked changes denoting where the text has been changed; the other a clean version (uploaded as the manuscript file).

Important additional instructions are given below your reviewer comments.

Please prepare and submit your revised manuscript within 60 days. If you anticipate any delay, please let us know the expected resubmission date by replying to this email. Please note that revised manuscripts received after the 60-day due date may require evaluation and peer review similar to newly submitted manuscripts.

Thank you again for your submission. We hope that our editorial process has been constructive so far, and we welcome your feedback at any time. Please don't hesitate to contact us if you have any questions or comments.

Sincerely,

Adrian M Haith
Associate Editor
PLOS Computational Biology

Samuel Gershman
Deputy Editor
PLOS Computational Biology

****

Dear Adrian and Samuel,

Thank-you very much for the comments and reviews. We have extensively revised the manuscript, taking in all of the comments of the reviewers, and adding additional Supporting Information to deal with several of the questions. We have also included all of the previous papers mentioned by reviewer 2, and explain how our work is different from these earlier studies. Importantly, in our manuscript we not only show that the human behaviour is consistent with two independent controllers, but also that it cannot be explained by one controller. Overall, we think that this has strengthened our manuscript, and helped us support our claims.

Sincerely,
Justinas Česonis and David Franklin

Reviewer #1: This study has demonstrated that the motor system can flexibly recruit different feedback controllers depending on the type of task (hitting and stop tasks). It seems that previous studies have already demonstrated this. However, the authors pointed out that this was not true: The identical feedback controller could produce different feedback responses, and therefore observing different feedback responses is not solid evidence for the recruitment of different feedback controllers. Interestingly, their previous study (Cesonis and Franklin 2020) had demonstrated that the identical feedback controller induced the identical feedback responses when they were examined at the same time-to-target.

They experimentally demonstrated that different feedback responses were induced for the hitting and reaching tasks, even when the responses were evaluated at the same time-to-target and that this result was reproduced by the OFC models with different cost functions for both tasks. Furthermore, the schedule of tasks (i.e., block or mixed schedule) did not influence the result, enabling them to conclude that the motor system can flexibly and rapidly switch between different feedback control policies.

It is well-known that the motor system can create and retrieve different feedforward controllers when the appropriate cue is provided. This study is a significant contribution demonstrating that the motor system also has the ability to switch between different control policies depending on conditions. I was also intrigued by the idea that the feedback response at the same time-to-target can be used as a probe that examined the feedback controllers. Since this study was well-designed, I only have a few very minor issues.

Response: We thank the Reviewer for the positive comments. We have revised the paper according to your suggestions.

1. Long-stop condition: This is an important control condition. The simulation result was presented, but the experiment was not performed in this condition. Do the authors expect that the experimental result would match the simulation?

Response: Kinematically long-stop and hit conditions would look very similar in the experiments, with the only difference being in the instruction. Here we worked under the assumption that even if participants are instructed to hit the target, they might reimagine the hit task as a long-stop condition, as it is effectively the same movement. As a result, our simulations of long-stop condition served not as a separate condition, but as a control check that participants indeed performed hit movements, rather than re-formulating the task in the hit condition. Therefore, while our results altogether lead us to believe that under the specific instruction the experimental long-stop condition would produce similar results to the simulation, this comparison was not our primary focus, and therefore we did not perform this condition experimentally.

2. Effect of schedule: I understood that there were no systematic differences between the blocked and mixed schedule (Fig.4). I do not oppose the idea of rapid switching, but I am

curious about if the aftereffect from one condition to another did not exist. In the mixed schedule in which the hitting and stop tasks were randomly changed, one condition should continue several trials, which might obscure the effect of interference. The difference could be observed when analyzing the data in the trials only after switching?

Response: We took deliberate care in pseudo-randomising the trial order in order to avoid condition clustering as much as possible. To do so, we did not randomise the trials for the entirety of the experiment, but rather split them into blocks and shuffled within. As a result, in a block of 32 trials in the Mixed schedule we always had an equal number of trials from both conditions. However, we acknowledge that consecutive trials of the same condition must have occurred. Indeed, on average in a mixed schedule, each participant saw 297 perturbation trials after the condition switch, and 275 perturbation trials with the same condition as in the trial before. We thus performed a follow-up analysis based on the reviewer's suggestion, which is now included as Supporting Information. However, both in trials that occurred after the condition switching, as well as in trials with the same condition as in the previous trial, we observe the same systematic variability.

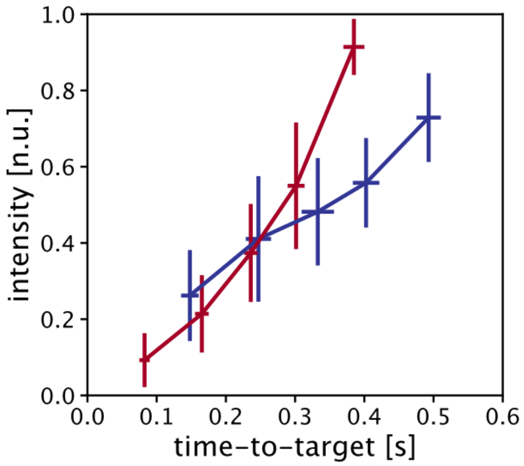

3. Fig.2.B, C and 3E, F: The intensity was likely to be calculated as an averaged feedback response (i.e., force) in 180ms and 230ms from the perturbation onset. There seemed some inconsistencies between Fig.2 and 3. The force output shown in Fig.2 was approximately 0.3N for the earliest perturbation, while the intensity in Fig.3 was 0.8N. Furthermore, the difference in the force output between both tasks (Fig.2B and C) is much greater than the difference in the intensity (Fig.3E, F).

Response: The two panels in Figure 2 aim at demonstrating relative variability of force evolution during the time after the perturbation. Here we display the raw data from our experiments, with forces that were actually recorded. In Figure 3 and later, in order to avoid biases towards the participants with stronger responses, we normalised the feedback intensities for each participant between 0 and 1. Hence the Figure 3 (and Figure 4) demonstrate normalised responses. We now make this clear in the figure captions and by renaming figure axes where appropriate.

4. line 117-118 on page 9: There are garbled characters before 1cm/s and 20cm/s.

Response: Corrected.

5. Line 356 on page 12: “In a recent study” should be “A recent study”?

Response: Corrected.

Reviewer #2: The paper by Cesonis and Franklin presents data about changes in control policies between trials that differed based on their instructions. In one set of trials, participants were instructed to reach and stop at a target. In another set of trials, they were instructed to hit the target. Based on the premise that these different instructions translate into flexible changes in control strategies, it is shown that participants indeed switched controller from one trial to the next. The paper is interesting and all technical aspects seem to have been performed well. But it its current form I cannot endorse the paper because it ignores or dismisses previous work on the same topic.

As is, the paper cannot be accepted because it is not novel. A first point is the switching of controllers across trials, which has long been known. In fact, it has been included in the experimental design of many previous studies. Without putting emphasis on it, Nashed and colleagues (2012) already analysed the difference between blocked and random design to highlight trial-by-trial changes in goal structure. Later, Lowrey and colleagues (J Neurophysiol, 117: 1070–1083, 2017) used a random design with trials towards a round target or a bar target randomly interleaved and highlighted task-dependent switching of controllers. Orban de Xivry (PloS one 8 (6), e66013) compared switching of controllers with a blocked or random schedule and found a switch albeit smaller. The switching per se is not new.

Response: We thank the Reviewer for the comments. We believe that implementing these and the following suggestions have improved our manuscript. We have now revised our introduction to include the above mentioned studies, as well as discussed their results in context of our study. Please see the highlighted changes in our Introduction and Discussion.

More striking was the effort to minimize our recent study investigating switching of controllers across and within trials by de Comité et al. (125 (5), 1883-1898), which again showed the same results. I find that the authors dismissed our previous findings. First, we included also a change in controller across trials in the absence of online perturbation of the target shape as a control condition, reproducing the results of Nashed, Lowrey and colleagues. Then, it is written “the change in control signals alone does not directly imply a change in the feedback controller”. Yet, in this previous study, the change in control was evoked by a change in the structure, not the state (for instance position and velocity) of the goal. The authors then argue vaguely that this change in control can result from a change in the state that includes the target representation. The statement is inaccurate at best, or misleading if intentional. I believe the authors have missed that, computationally, changing the structure of the target from a square to a bar corresponded to a change in task demand. The critique was unfair and the author did not give proper credit to this previous work.

Towards assessing the paper as objectively as possible, it must be recognized that computationally again, our previous experiment corresponded to manipulating the factor called wp,t in their equation (3), which either has two non-zero components (penalizing x, and y) or one (penalizing only y). What the authors have done is testing the same idea by manipulating the other factors weighting velocity and force in the cost function. It is an important and interesting contribution on its own, but it does test the same idea only with other state variables.

As such it deserves publication provided that previous contributions are properly acknowledged. The change of controllers has been demonstrated before, this is not new, what is tested here is whether this result also applies to hit and stop conditions, which are believed to require a change in the controller not linked to target geometry but to the penalty on the final state. Both manipulations imply a change in cost-function that must translate into an update of feedback gains. A substantial reworking is necessary to make this aspect very clear and upfront.

Response: we have reworked our Discussion paragraph, discussing the aforementioned study (lines 364-392 in the revised manuscript). We hope it is now clear that our aim is to build on earlier work, particularly in the areas where we feel there was leeway for interpretation, as opposed to attempting to diminish the quality of work that shows interesting results.

Specific points:
I did not find any definition of the time-to-target in the data, apologies if I missed it. Since there is no variability in time-to-target of Fig. 4b, I am assuming that it is measured as the difference between the perturbation time and some time limit, but since the perturbation were triggered in position I failed to understand how there was no variability in this quantity.

Response: The Reviewer is correct – the time-to-target is a variable quantity. We now explicitly define time-to-target in lines 41-42, and added errorbars to the figures where the time-to-target is evaluated.

The authors equate urgency and time-to-target, which is a bit hazardous because the urgency may refer to an instantaneous pressure signal, without predicting how long or how far in time will movements be completed.

Response: We have slightly rephrased this sentence. Instead of equating time-to-target with urgency directly we now mention it as one aspect of urgency.

With respect to computational modelling I had concerns about the comparison made between the forces measured experimentally in a force channel and the forces produced in simulations. Was there a simulation of a force channel? Is there an impact on the correspondence between measured and simulated variables? Pls. add justification to the paper.

Response: The original simulations were not performed in the channel trials for a few reasons. First, we are interested in modelling and testing the actual control behaviour that is unconstrained. While we tested the human participants in channel trials, the motor response produced by humans is not directly affected by the feedback of the channel due to the specific timing of the feedback window (180-230 ms) and delays associated with the visuomotor feedback loop (~150 ms from visual stimulus to force production). As the first responses happen at ~150 ms after the perturbation, participants would only produce different force responses at ~300 ms if they notice that the cursor is not laterally responding to their control input. Any stretch response effects can also be ruled out in the time window of 180-230 ms, as described in Dimitriou et al 2013.

The major difference where the channel trials affect the movement differently than free movements with maintained perturbations is in the movement timing. Here in our simulations we kept the timing consistent with the channel trials, so that our simulation results are representative of the required movements.

In addition, we expanded our simulation code (provided as supplementary material) to simulate movements in the channels (see Figure below) and Supporting Information. The produced behaviour is qualitatively indistinguishable and we discuss this at lines 498-506 of the revised manuscript.

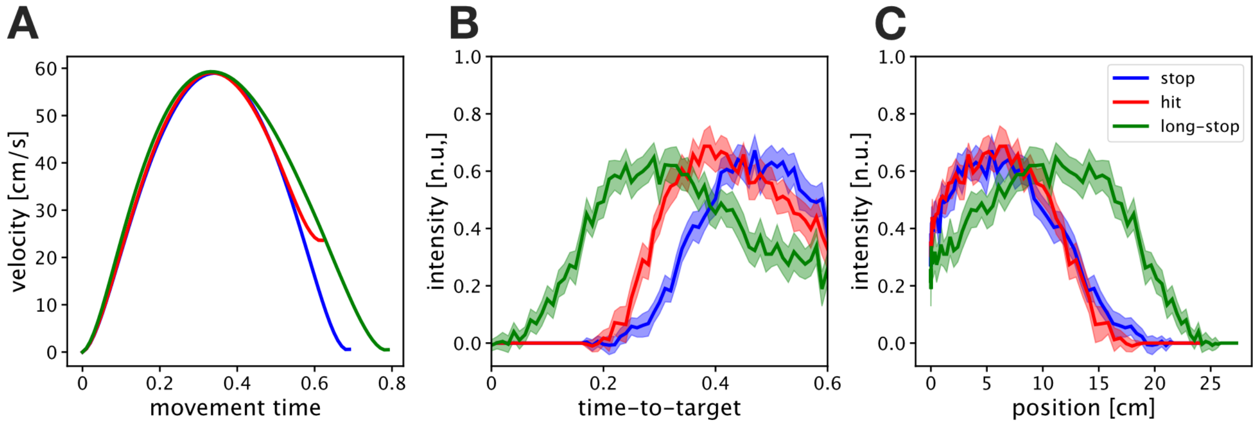

I found that the overall narrative was sometimes unclear. It has been known for a long time that between blocks of trials with different instructions, humans can change their goal-directed policy. This in my view is the main contribution of the use of optimal feedback control. In contrast, in the literature on motor adaptation shows gradual changes in internal models in ABA learning designs, with unlearning and interferences. Thus, it seemed clear that internal models for reaching do not change as quickly as task-dependent policies. The case of motor adaptation requires special contextual cues that seem less important for task-related constraints. The analogy is also limited, as context-dependent motor adaptation is associated with different feedforward controllers, while in the case of change in task-demands the authors use a model without feedforward pass. If the results must be interpreted in light of context-dependent learning I admit I see more differences between these paradigms than what is suggested.

Response: First, we have specifically chosen movements and tasks that should already have existing controllers, partially because they are so simple (reaching and hitting) and partially because we have done these tasks over our entire life. If we chose truly novel tasks, we expect that we could see learning of these feedback controllers: slow changes over trials. Importantly these effects have been clearly seen, where learning the appropriate feedback controller for new tasks such as mirror reversal is only slowly produced. On the other hand, dynamics that we have learned over our entire lives should also be instantly switched between just as the feedback controllers are. Hence the fast or slow development of control, independently of feedforward or feedback, is a result of familiarity with the task, rather than something inherent to the type of control.

Respectfully submitted,
F. Crevecoeur

Reviewer #3: In this paper the authors investigate to what extent healthy participants can switch between feedback controllers across tasks. This issue is clearly of interest to the motor control community. Indeed, although previous work has shown that task-dependant switching of feedforward controllers was possible, to my knowledge this issue has never been addressed in the context of feedback controllers. By comparing how participants react to visual perturbations (i.e. unexpected lateral target jumps) when performing either a reaching (stop) or an intercepting (hit) task, the authors can probe that these two tasks rely on separate feedback controllers. Importantly, evidence for separate controllers can be found even when participants are required to alternate reaching and intercepting trials. The authors conclude that contextual switching of feedback controllers is possible (as previously observed for feedforward controllers). In general, I found the manuscript very well written, with clear objectives, using a careful/clever design, and offering a lot of technical details. Still, I believe that this manuscript can be improved in several places (see my comments below).

Response: We thank the Reviewer for positive comments about our manuscript. We have revised the text and think that this has improved the strength of our claims.

Major Comments

1) Methodology (line 593-596): The authors state that all participants (n=14) performed first the blocked-design conditions then performed the mixed-design conditions (alternation of tasks). I wonder to what extent this design has favored the emergence of separate feedback controllers. When a participant performs a block of 416 trials while executing the same task over and over, it seems adequate to develop a feedback controller that is specific to this task. When the participant is subsequently asked to alternate trials in one task with trials in the second task, one may assume that those preexisting (separate) controllers may facilitate the switching of controllers across tasks. Now it is less clear what would have happened if participants had been exposed first to the mixed-design schedule (rapid alternation of tasks). One may wonder whether the emergence of separate controllers would have been possible. Although, I feel confident with the observations reported by the authors, I believe this extra information would impact the extent of the conclusions formulated by the authors. Obviously, I understand that investigating this issue necessitate to collect extra participants (but one may argue that the current number of participants is on the low side), but if such material is not provided, the authors should at least acknowledge the limitations of their experimental design.

Response: In this study we tested whether the switching of feedback controllers could be present in humans when rapidly switching between two tasks. In our experimental design we consciously aimed to select two tasks (hit or stop) that were not novel to our participants. The reason for such choice was to simulate as close to real-life conditions, where humans are already skilled in performing a given task in isolation (i.e. already have pre-existing controllers, dedicated to that task), before being required to alternate between multiple tasks. However, there remains a possibility that due to the dynamics of the vBot environment both tasks were novel to participants. As a result, we always started our studies with the blocked schedule, and only then followed with the mixed schedule. An interesting control would be to first test the

participants in the mixed schedule, followed by the blocked schedule. However, we believe such control would mainly test whether the two task choices were novel to participants or not, which is not the focal point of our study. We have added this point to our discussion of possible limitations (lines 506-516 in the revised manuscript).

2) Data analysis: In Fig 4B feedback response intensities are provided in each task (hit and stop) and for each schedule (blocked vs. mixed). Visual inspection suggests that the difference across tasks is greater in the blocked design (red vs. dark blue curve) than in the mixed design (orange vs. light blue). I wonder if the authors performed some statistical analyses that addressed specifically this issue (apart from the lack of condition/schedule interaction), as a smaller disparity across controllers matches with one the hypotheses formulated initially. One would also appreciate further analyses of the mixed schedule to assess the presence of possible carry-over effects when switching across tasks. For instance, one could assess whether the gain of feedback responses in a stop/reach trial increases as the number of preceding hit/intercepting trials increases. A possible lack of carry-over effects across tasks would strengthen the conclusions made by the authors (i.e. fully independent feedback controllers allowing instantaneous switch).

Response: We did not perform other additional tests other than the two-way ANCOVA to compare our four conditions, as we strongly believe this is the most appropriate way to make these comparisons. However, we understand that in a conventional frequentist analysis the lack of effect (as in the case of condition/schedule interaction) could come not only from true lack of differences, but also from low testing power. To account for this, we also included Bayesian versions of all the tests that we performed, as such analysis has three decision regions (effect, no effect, and "in between"). As the interaction term in Bayesian ANCOVA shows tendency towards no effect, we trust the results of our statistical analysis.

On average in a mixed schedule, each participant saw 297 perturbation trials after the condition switch, and 275 perturbation trials with the same condition as in the trial before. As a result, in order to keep the power of the follow-up analysis higher we compared the intensities in trials that followed a condition switch against the trials that followed the trial of the same condition. This analysis is now included as a Supporting Information. However, both in trials that occurred after the condition switching, as well as in trials with the same condition as on the previous trial, the same systematic variability is observed as was found in the blocked schedule, and as was found in the mixed schedule as a whole.

3) Learning: As far I understand, all available (perturbed) trials were considered to assess the visuofeedback gains associated with each task. It is unclear to what extent these responses are modulated by prior experience with the task (and perturbation). It would be helpful to clarify whether these feedback controllers were preexisting/innate, or whether they slowly build up during practice with the task. One could imagine that during initial training, feedback controllers were rather similar for both tasks, but progressively diverged with further experience. Providing some information about the time course of feedback responses over the blocked schedule would be helpful. For instance, one could contrast responses in early and late trials. Obviously, I

understand that the statistical power will drop, but clarifying how these feedback controllers appeared/emerged in the first placed seems critical.

Response: As we state in a new Discussion paragraph we intended to select tasks with pre-existing controllers so that we could directly test for their switching. However, there remains a possibility that due to the dynamics of the vBot environment both tasks were novel to participants. We performed an additional analysis on the blocked schedule, where participants first experienced these two tasks, and looked at the development of feedback intensity profiles within the first 3 blocks. Importantly, even in these initial few blocks the controllers are producing distinct responses to hit and stop tasks, suggesting that the controllers were innate to our participants (or developed so rapidly, that for all intents and purposes in this study these controllers could be considered innate). We have included these results in Supporting Information and added discussion of these points to the manuscript.

Minor comments

Line 117-118: Remove the extra characters preceding the value of velocity

Response: Corrected

Line 330: Please consider that not all studies support the notion that feedback and feedforward mechanisms are necessarily shared (Abdelghani and Tweed 2010; Gritsenko and Kalaska 2010; Kasuga et al. 2015; Yousif and Diedrichsen 2012).

Response: We have added the Reviewer's suggestions to Discussion (lines 349-352 in the revised manuscript) where we mention that some studies indeed suggest feedforward and feedback controllers use different neural circuits.

Line 418: The authors emphasized that "time-to-target" is a better reference frame to monitor feedback responses. This variable resonates with another variable "time-to-contact" (Tau) that is very popular in the field of ecological psychology. I am wondering if the authors considered a possible connection with this field, or this is just a fortuitous coincidence?

Response: The answer to this question is two-fold. On the one hand, time-to-target reference frame is an outcome of the mathematics of the finite-horizon LQR (or LQG) controller, where the cost-to-go is calculated for the movement, starting from the movement end and going towards the beginning. On the other hand, for such an algorithm to be possibly applied within the human motor system, a variable like time-to-target or time-to-contact must be accurately estimated. In our previous work (Česonis & Franklin 2020) we indeed discussed time-to-contact as a phenomenon, enabling such control.

Line 505: Was there a rational for the number of participants (n=14)? Nowadays, more and more studies are considering sample size of at least 20 participants.

Response: While we agree that the number of participants in our study is on a lower end, a couple of factors lead to this decision. First, while recent studies indeed consider sample sizes of 20 or more participants, typically tasks are also shorter (500 - 800 trials, compared to 1664 in our study). Due to the nature of the task, we can thus reduce individual noise of our participants by performing more observations per participant. For example, here we collect 52 repeated measurements for the same type of perturbation for each participant, whereas within a typical learning task where the response evolution over time is important, only 1 measurement per participant would be included into the population mean.
Second, typically visuomotor responses produce a strong effect of the phenomenon. Indeed, similar studies (Dimitriou et al 2013, Franklin et al 2016) are able to demonstrate the effects within a single subject, thus producing very reliable data.
Finally, contact restrictions in Germany due to COVID at the time of the manuscript preparation also significantly limited the participant pool.

Line 536: In the intercepting task, people were given feedback to hit the target at a given speed, but were they given explicit instructions regarding the fact that their peak velocity had to coincide or not with the hit?

Response: Participants were given full instructions regarding their movement kinematics, as described in the Methods lines. 613-615 in the original manuscript. They were required to produce movements of peak velocity at a specific magnitude and position (as also shown in Fig 5D), which was matched across both conditions. As a result, following these instructions resulted in velocity requirements where the hit was performed at a lower than peak velocity, but significantly faster than in the stop condition. Importantly there was no requirement to hit the target with a specific speed, although the requirements regarding distance and peak velocity make the velocity at the time of target impact consistent across participants.

Line 598: Did you have any constraints regarding the randomization of task order? For instance, was it possible for participants to experience 3 (or possibly more) consecutive trials of the same task? Did you set a limit for this (i.e. maximum number of consecutive trials in the same task)? More information on this issue would be helpful in the method.

Response: We divided trials into blocks, where each block contained each different perturbation trial exactly once, and also 5 free movements of each type. That is, within the blocked schedule each block contained 16 trials (11 perturbed + 5 free), and within the mixed schedule each block contains 32 trials (16 of each condition). Within the block, we freely shuffle the order of the trials, meaning that in the mixed schedule, for every 32 trials, there will always be 16 trials of each condition. While this still leaves the random chance of some trial clustering, over 52% trials were presented after the switch in condition, 26% after exactly one trial of the same condition, and 12% after exactly 2 trials of the same condition, with larger clusters in diminishing quantity. We added this information to the Methods (lines 634-638).

Line 631: I assume that the neutral force profile was obtained from the non-perturbed trials performed with the channel, correct? I presume this force profile was non monotonic (possibly

velocity dependent), correct? How did you deal with the fact that movement time varied across trials?

Response: all of the Reviewer's observations are indeed correct. As a result, the feedback intensities in individual trials are very noisy. However, for each displayed datapoint we collected 52 measurements for each participant (26 blocks x 2 directions) to limit this noise as much as possible.

Line 637: Although I do understand the rational for the normalization of force responses across participants, it does not seem to match with the unit employed in Figure 3 and 4. In both figures the intensity of feedback responses is presented in Newtons. Please clarify how the normalization procedure allows the preservation of feedback responses in Newtons.

Response: We have replaced Newtons [N] by the normalised units [n.u.] in the figures where normalised results are presented.

Fig 1: The authors contrast two extreme situations: a single versus 2 independent controllers. One could imagine an intermediate scenario in which the single controller carries history dependent effect (see also my second main comment on this). Perhaps the authors could acknowledge this third option in the caption or somewhere in the text.

Response: Our single controller is exactly what Reviewer describes. It has a history dependent effect that would gradually tune itself to whatever environment/task is present. This is why we would predict differences in the blocked design.

Fig 3A&D: For both tasks, peak velocity is about 10% smaller in the experimental data than in the model predictions (60 cm/s). Even if the predictions would probably be qualitatively similar, I wonder why the authors did not readjust the settings for the model predictions.

Response: We chose such model settings primarily as we wanted to set the same instructions for our simulations as we did for our participants. For example, a low-end peak velocity could also be a systematic motor behaviour arising from the given task constraints. However, the reason for the discrepancy between the values in the figures is not due to the lower produced velocities by our participants, but rather due to the temporal trace averaging: the mean peak velocities over the course of the whole sample were ~58.5 cm/s, however on individual trials the timing of this peak varied a little. As we averaged the whole traces to display mean kinematics, such averaging tends to produce lower values overall (see illustration below as an example). We now also report the mean experimental peak velocities (line 176 in our revised manuscript).

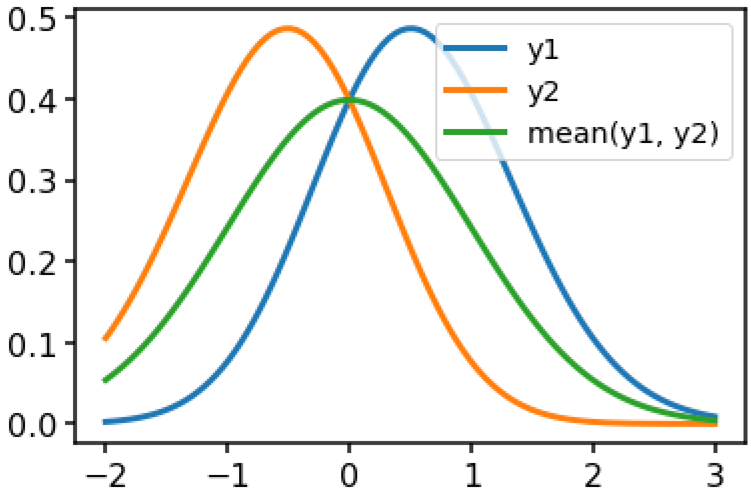

Figure 3B: It is a bit odd to provide a single label (B) to account for two graphs (time-to-target and time-to-movement end) as opposed to all the other graphs (one label per graph). I would suggest inserting an extra label for the neighboring graph (with final label reaching G instead of F).

Response: We have relabelled the graphs based on Reviewer's suggestion

Fig 3BC: The model predictions suggest that sometimes the intensity of feedback responses can be negative (below 0). This feature is not visible in the experimental data (see Fig 3E) which presumably makes sense given the way the force data were analyzed (as force direction did not seem to matter). Altogether I am left wondering how to reconcile these separate observations. Please clarify.

Response: We included noise in our simulations, which could bring simulated feedback intensities to the opposite values, particularly when required corrections were low. We also observe such a phenomenon within individual responses in human participants, however it disappears with a large enough sample size (i.e. with increasing power), as the mean net corrections are against the perturbations. This perhaps suggests that our model noise is on the higher-end, however we did not deliberately aim to match noise to the experimental values. However, it is important also to clarify that force direction is taken into account in our analysis - so that positive values are always in the direction to counteract the visual perturbation. This is mentioned in the Data Analysis section of our Methods (l 634-636 of the original manuscript; l 672-676 of the revised manuscript): "As the direction of the response differed based on the perturbation direction, we reversed the direction of the intensities of responses to the leftward perturbations and grouped all intensities by the perturbation onset location." We have now clarified this statement.

Fig 3E+Line 153: This graph clearly shows different feedback responses across tasks, and the authors capitalize on these differences. Apart from being consistent with optimal feedback

control, can the authors provide a (simple) rational as to why, during the first part of the movement, feedback responses should be larger for the hit condition than for the stop one?

Response: Many recent studies that our work builds upon suggest that humans perform reaching movements that are close to optimal. While this hypothesis still has limitations in some areas, it allows for some powerful predictions on human behaviour. Here we believe that humans produce stronger responses for the hit condition than for the stop in the early part of the movement simply because such responses are optimal under the given conditions. However, the particular intensity profiles are only specific to our task, as we carefully matched the experimental conditions to the model settings. Different duration of the reaching movement, different velocity conditions or distance would predict a different regulation than shown here. The fact that hit responses are stronger at the beginning of the movement is not a universal phenomenon, but a specific outcome of our design, which is useful when observing the separation of the two controllers.
