# Supplementary material for: Contextual cues are not unique for motor learning: Task-dependant switching of feedback controllers
Source: PLoS Comput Biol. 2022 Jun 9;18(6):e1010192. doi: 10.1371/journal.pcbi.1010192 (PMC9217135; doi:10.1371/journal.pcbi.1010192)
Supplement: Attachment — Submitted filename: response_to-reviewers.pdf [file pcbi.1010192.s005.pdf]

Dear Mr Česonis,
Thank you very much for submitting your manuscript "Contextual cues are not unique for motor learning: Task-dependant switching of feedback controllers" for consideration at PLOS Computational Biology. As with all papers reviewed by the journal, your manuscript was reviewed by members of the editorial board and by several independent reviewers. The reviewers appreciated the attention to an important topic. Based on the reviews, we are likely to accept this manuscript for publication, providing that you modify the manuscript according to the review recommendations.

The reviewers were generally very satisfied with the revisions to the manuscript. Reviewer 2 raised some remaining concerns, however. I don't think these are major issues, but I hope you might be able to address them (as well as those raised by Reviewer 3) by providing a response to the reviewers' concerns and revisions to the manuscript where these may be appropriate.

Please prepare and submit your revised manuscript within 30 days. If you anticipate any delay, please let us know the expected resubmission date by replying to this email.

When you are ready to resubmit, please upload the following:

[1] A letter containing a detailed list of your responses to all review comments, and a description of the changes you have made in the manuscript. Please note while forming your response, if your article is accepted, you may have the opportunity to make the peer review history publicly available. The record will include editor decision letters (with reviews) and your responses to reviewer comments. If eligible, we will contact you to opt in or out

[2] Two versions of the revised manuscript: one with either highlights or tracked changes denoting where the text has been changed; the other a clean version (uploaded as the manuscript file).

Important additional instructions are given below your reviewer comments.

Thank you again for your submission to our journal. We hope that our editorial process has been constructive so far, and we welcome your feedback at any time. Please don't hesitate to contact us if you have any questions or comments.

Sincerely,

Adrian M Haith
Associate Editor
PLOS Computational Biology

Samuel Gershman
Deputy Editor
PLOS Computational Biology

Dear Adrian and Samuel,

Thank-you very much once again for the comments and reviews. We have now finalised our manuscript, based on the remaining feedback from Reviewers 2 and 3. We hope that this version fully addresses any outstanding questions, and we want to thank you and the Reviewers for the helpful and constructive review process.

Sincerely,
Justinas Česonis and David Franklin

Comments to the Authors:

Reviewer #1: I was satisfied with the revision. I have no further additional comments.

Response: We thank the Reviewer.

Reviewer #2: The authors have clarified their contribution in regard of previous work investigating changes in target structure and the paper has improved. There remain points of disagreement that I would recommend the authors to consider.

Response: We thank the reviewer for their helpful comments. We have revised the manuscript as described below.

Regarding the impact of the channel I am surprised to see the arguments developed, visuomotor delays are closer to 100ms than 150ms, see for instance Izawa and Shadmehr (JNS, 2008) who documented the effect of a jump on acceleration. Regarding the stretch response, the statement seems inaccurate in my view because the smallest perturbation loads applied very early during reaching are known to evoke short and long-latency responses (J Neurophysiol, 2012, 107: 2821-2832). In all it seems that there can be a feedback-mediated response to the channel, which is not captured in the simulation since there was no difference with or without channel. I believe that this discrepancy between model and data could be expanded a bit. It is strange to argue that the experiments aimed at probing control while using channels, then use a model without channel to simulate control and verify that results in the channel are not different.

Response: Multiple points can be isolated in the Reviewer's comment. First, we need to correct our phrasing in our previous response to Reviewer's comment on this topic: the stretch reflex could indeed be evoked by the general presence of the channel, and as mentioned by the reviewer, even small loads can elicit both short and long latency feedback responses (J Neurophysiol, 2012, 107: 2821-2832). However, the onset of the channel occurs prior to any movement of the participant, long before the time of the visual perturbation, and so will not suddenly produce a stretch reflex response timed to the visual stimuli. More importantly, these effects would be consistently present in all channel trials, including zero-perturbation, as well as be independent of the visual perturbation direction. As we subtract zero-perturbation force traces to obtain the net response to visual perturbations in the pre-processing stage, such effects of the stretch reflex would have no influence on the net visuomotor correction.

We have included further details of these points in the discussion. Specifically we state:

"Another possible limitation of using channel trials to probe the feedback control is the potential interference of the stretch reflex. Specifically, small forces produced on the hand by the channel could set on the feedback corrections (Crevecoeur et al 2012) that superimposed onto the measured visuomotor responses. However, as the onset of the force channel occurs prior to any movement of the participant, long before the time of the visual perturbation, the channel onset will not produce a stretch reflex response timed to the visual stimuli. More importantly, such effects, as well as any other corrections to the channel onset would be present in all channel trials (including zero-perturbation trials), and thus would cancel-out in the net feedback responses, as they would not depend on the direction of the visual perturbation. As a result, it is unlikely that the force channels introduced systematic effects into our recorded visuomotor feedback signal. Similarly, we also saw no behaviour differences between simulations of free movements, that we presented in our results, and analogous simulations of matching duration movements in channel trials (S2 Text)."

While the reviewer mentions one paper that found a visuomotor delay of around 100 ms (Izawa and Shadmehr, 2008), we have conducted many detailed experiments carefully measuring the onset of the visuomotor feedback response with this system and these specific perturbations, showing that the onset in terms of changes in force occurs around 150 ms (for example: Franklin et al., 2008; Dimitriou et al., 2012; Franklin et al., 2014; Franklin et al., 2016; Franklin et al., 2017). In particular, I would like to draw the reviewers attention to our paper which uses 20 participants performing four days worth of experiments to determine the visuomotor feedback onset timing (Franklin et al., 2016), finding values of about 146 ms.

I did not follow the authors' response regarding the difference between change in control and adaptation, and I did not see any addition to the manuscript about this point. An indirect argument is made about the fact that the task was not novel but to my knowledge, dual adaptation can be compromised during exposures longer than the training sessions used here. In addition, the authors suggest that it would be an interesting control to test first the random schedule. Indeed, and I would be willing to bet that it works just fine or to a slightly lesser extent. On the contrary dual adaptation builds up over lover periods of time, and visual cues (and others) have been shown ineffective in evoking different contexts or goals. These are clear differences and I suggest to discuss the limitations of the analogy with context-dependent adaptation in more detail.

Response: We thank the reviewer for this comment. First, a distinction needs to be made between contextual switching and motor adaptation. Typically, studies of dual adaptation utilise contextual cues to separate different contexts in which two (or more) different tasks are learnt. In these studies participants are usually given novel tasks, for which they do not have any pre-existing motor memories (e.g. curl force fields), and thus slow adaptation is observed. However, if the task is familiar (e.g. participants have learned these curl fields in exact same contexts) we will not see extended adaptation and adequate force compensation will be produced almost immediately. As an analogy, this is why we do not expect a tennis player to learn the dynamics of the racket every time they come to play the game. Such instant switching can be seen even in force fields after the first day of training - see for example the clear switching responses in the fifth day (Figure 3) of Howard et al., 2015, Current Biology, and on the second day of experiments in (Figure 3) of Howard et al., 2020, PloS One.

Our task that we provided in our study is a simple reaching (or "punching") movement, which is likely not novel for participants, therefore it is reasonable to expect that the controllers for such tasks are pre-existing. As a result, it similarly shouldn't be surprising that the appropriate feedback controller is used for a task without a long training with gradual adaptation; we do not claim dual adaptation, but we believe that our results demonstrate contextual switching.

Whether we can evoke contextual switching in the first place is what we aimed to test in our study. If this is not possible, in our study we expected to see both hit and stop movements to be executed with the same feedback controller (whether the average for both movements, or any of the individual controllers). We would expect similar behaviour if feedforward controllers can't be separated.

As Reviewer states, visual cues have previously been shown to be not effective in separating different contexts (although note that visual cues of objects have shown to separate contexts partially, with adaptation up to 40% within one training session (Figure 6, Howard et al., 2013, J Neurophysiol) and visual lead-in movements show very strong separation (Howard et al., 2012, J Neurosci)). However we do not claim that the visual cues

(target colour or the general appearance of the workspace) directly regulated the contextual switching. Instead, we believe that the controller switching was induced by different task goals, associated with each workspace. Similar regulation has also been shown in feedforward control (Sheahan et al, 2016, Neuron).

We have revised the discussion to add these points to clarify our position. Specifically we state:

"Most studies of motor learning study contextual switching in conjunction with dual adaptation by introducing participants to novel force fields or visuomotor rotations, for which they do not have pre-existing feedforward controllers. In turn, we typically see slow, simultaneous adaptation to applied perturbations, as well as context-induced switching of the memories after the transient learning phase is over. Importantly, for familiar tasks this switching is evoked immediately, without the need to re-learn the dynamics again on re-exposure. This is clearly seen on the second or later days after adapting to dual force fields Howard et al 2015, Howard et al 2020. In this study, our main goal was to demonstrate that such contextual switching is also possible for feedback controllers, rather than to demonstrate gradual adaptation. Therefore, in our experimental design we consciously selected two tasks (stop or hit) that were not novel to our participants."

Reviewer #3: I am generally satisfied with the responses/changes provided by the authors to my concerns. Specifically, I appreciated their effort to assess the presence of possible carry-over effects across tasks. Evidence for instantaneous switching is strengthened. I have only a few minor suggestions left to improve their manuscript.

Response: We thank the reviewer for their helpful comments. We have revised the manuscript as detailed.

1) Fig S1: Please state the nature of error bars (SEM or 95% confidence interval)

Response: corrected (95% CI)

2) Fig S2: It I unclear why data on panel B is displayed in arbitrary unit, whereas it is displayed in raw unit (N) in panel C. The resolution of the Y axis in panel C is low compared to other panels (2 versus 6 values). Please accommodate graph C accordingly. As evoked previously for Fig S1, please state the nature of error bars (SEM or 95% confidence interval).

Response: Panels B and C show the same data, however the data in panel B is normalised, while in panel C the raw data was used to generate the plot. As we used only a subset of our data in S2, these results are sensitive to noise, and thus the normalisation could be affected by an occasional outlier. As a result, for completeness, and a clearer picture, we wanted to present both normalised data (panel B), and raw data (panel C). We have added this information to the S2 Text.
We changed the resolution in panel C, and specified the error bars (95% CI).
